# Development of Structure-Switching Aptamers for Kanamycin Detection Based on Fluorescence Resonance Energy Transfer

**DOI:** 10.3389/fchem.2019.00029

**Published:** 2019-02-07

**Authors:** Xinyue Ma, Shangna Qiao, Hongjing Sun, Ruifang Su, Chunyan Sun, Mingdi Zhang

**Affiliations:** ^1^Department of Food Quality and Safety, College of Food Science and Engineering, Jilin University, Changchun, China; ^2^Department of Food Science and Engineering, College of Food Science and Engineering, Jilin University, Changchun, China

**Keywords:** aptamer, structure-switching, fluorescence, FRET, kanamycin

## Abstract

The structure-switching aptamers are designed for the simple and rapid detection of kanamycin based on the signal transduction principle of fluorescence resonance energy transfer (FRET). The structure switch is composed of kanamycin-binding aptamers and the complementary strands, respectively labeled with fluorophore and quencher, denoted as FDNA and QDNA. In the absence of kanamycin, FDNA and QDNA form the double helix structure through the complementary pairing of bases. The fluorophore and the quencher are brought into close proximity, which results in the fluorescence quenching because of the FRET mechanism. In the presence of kanamycin, the FDNA specifically bind to the target due to the high affinity of aptamers, and the QDNA are dissociated. The specific recognition between aptamers and kanamycin will obstruct the formation of structure switch and reduce the efficiency of FRET between FDNA and QDNA, thus leading to the fluorescence enhancement. Therefore, based on the structure-switching aptamers, a simple fluorescent assay for rapid detection of kanamycin was developed. Under optimal conditions, there was a good linear relationship between kanamycin concentration and the fluorescence signal recovery. The linear range of this method in milk samples was 100–600 nM with the detection limit of 13.52 nM (*3*σ), which is well below the maximum residue limit (MRL) of kanamycin in milk. This method shows excellent selectivity for kanamycin over the other common antibiotics. The structure-switching aptamers have been successfully applied to the detection of kanamycin spiked in milk samples with the satisfying recoveries between 101.3 and 109.1%, which is well-consistent with the results from LC-MS/MS. Due to the outstanding advantages of facile operation, rapid detection, high sensitivity, excellent specificity, and low cost, the application and extension of this strategy for rapid determination of antibiotics in food samples may greatly improve the efficiency in food safety and quality supervision.

## Introduction

Kanamycin is an aminoglycoside antibiotic purified from *Streptomyces kanamyceticus*. Kanamycin is widely used to treat gram-negative and gram-positive infectious diseases in humans and veterinarians. Like other aminoglycoside antibiotics, kanamycin exhibits comparatively narrow safety margin, and the accumulation and enrichment of kanamycin in animal-derived foods can be caused if the dosage is not controlled (Turnidge, 2003). High doses of kanamycin can cause ototoxicity, nephrotoxicity and allergic reactions to the drug (Begg and Barclay, 1995). The maximum residue limit (MRL) of kanamycin in milk has been set in many countries and regions, for example, in European Union it is regulated to be 150 μg/kg, and in China 200 μg/kg. At present, the analytical methods for kanamycin mainly include instrumental analysis, immunoassay and biosensor, such as capillary electrophoresis (Kaale et al., 2003; El-Attug et al., 2011), high performance liquid chromatography (HPLC) (Chen et al., 2006; Blanchaert et al., 2013), enzyme-linked immune sorbent assay (ELISA) (Loomans et al., 2003), surface plasmon resonance (SPR) (Raz et al., 2009; Frasconi et al., 2010), and electrochemical immunosensor (Wei et al., 2012), optical aptasensor (Ha et al., 2017; Dehghani et al., 2018; Liu et al., 2018; Tang et al., 2018; Zhu et al., 2018), electrochemistry aptasensor (Sharma et al., 2017; Li et al., 2018), etc. But these detection methods show different advantages and disadvantages in practical application. The instrumental analysis has high sensitivity and leads to reliable results, but requires expensive equipment, high operational skills, tedious pretreatment and relatively long time, which makes it inconvenient for real-time analytics and a large number of samples. Immunoassay is fast and easy to operate, but antibody requires high cost and harsh preservation condition. The quality of different batch antibody may be different and the test may get false positive results. The aptasensors exhibit excellent performance, such as high sensitivity, easy operation, and better meet the needs for rapid detection. Therefore, it is very meaningful to establish a convenient, sensitive and low-cost method for rapid and efficient detection of kanamycin in complex food matrix.

Aptamer is a single-stranded DNA or RNA with a unique secondary structure or tertiary structure, which is obtained through exponential enrichment of ligand system evolution (SELEX) and can specifically identify the target (Ellington and Szostak, 1990; Tuerk and Gold, 1990). Due to the advantage that the aptamers can be obtained through *in vitro* screening, the application range of aptamers was greatly broadened without using animal *vivo* immunization. In principle, aptamers with high affinity can bind to any specific target, from small molecules to proteins or even more complex targets, such as metal ions (Rajendran and Ellington, 2008; Babamiri et al., 2018), small organic molecules (Dehghani et al., 2018), amino acids (Harada and Frankel, 1995; Zhu et al., 2011), proteins (Kim et al., 2018), cells (Zhang et al., 2018), and bacteria (Srinivasan et al., 2018), etc., making it possible to develop aptamer-based biosensors for a wider range of applications. Compared with traditional antibodies, the aptamers have the advantages of low cost, good thermal stability, easy production and modification. Thus, the aptamers have a good prospect in food safety monitoring and can be widely used in the detection of toxic and hazardous substances (Lv et al., 2016; Taghdisi et al., 2018).

In the aptamer-based biosensors, transducing aptamer-target recognition events into easily detectable signals is an important condition to realize the widely application of aptamers (Nutiu and Li, 2004). Therefore, a series of methods based on signaling aptamers have been proposed (Nutiu and Li, 2004), taking advantage of the fluorescence assay characteristics, such as direct observation, easily operation, good selectivity and high sensitivity. Jhaveri et al. (2000) reported a method, which involved aptamers labeled only with a single fluorophore, using the change of aptamer structure to achieve the quantitative detection for adenosine. Tyagi et al. (Tyagi and Kramer, 1996) designed a molecular beacon to detect specific nucleic acids, which involved a hairpin shaped oligonucleotide labeled with a fluorophore on one end and a quencher on the other end. These two methods have good detection performance for specific targets, but both of them need to consider the aptamers' key secondary or tertiary structures, which cannot meet the requirement of universal application. To overcome these shortcomings, the first structure-switching reporter based on a short sequence DNA aptamer was designed for ATP detection (Nutiu and Li, 2003), which is formed between a fluorophore-labeled DNA aptamer and a small oligonucleotide modified with a quenching moiety and function by switching structures from DNA/DNA duplex to DNA/target complex. In order to perfectly suit for any given aptamer, more strategies based on structure-switching aptamers have been designed and applied (Nutiu and Li, 2004).

The basic principle based on the structure-switching aptamers is the specific aptamer-target recognition and the fluorescence resonance energy transfer (FRET). The FRET refers to the transfer of electron excitation energy between an appropriate energy donor and an energy receptor pair, with a maximum transfer distance of 7–10 nm. Because of its extreme sensitivity to distance, the FRET method has been widely used to study the structure, properties, reaction mechanism and quantitative analysis of biological macromolecules (Okamoto and Sako, 2017; Chen et al., 2018). The structure-switching aptamers include the fluorophore-labeled and the quencher-labeled oligonucleotides, so the fluorescence is quenched when the fluorophore and quencher groups get close enough to each other. When the target is added, the aptamer binds specifically with the target, leaving the two groups far away and resulting in the fluorescence recovered. In recent years, more and more researchers are making efforts in this regard because of the simple, universal, specific and sensitive features of the structure-switching aptamers. Herein, the structure-switching aptamers based on the FRET are designed and used for kanamycin detection, and the results showed good sensitivity and selectivity.

## Materials and Methods

### Reagents

Kanamycin, chloramphenicol, streptomycin, tetracycline, and oxytetracycline were purchased from Shanghai Sangon Biological Engineering Technology & Services Co., Ltd. (Shanghai, China). Ciprofloxacin was purchased from Sigma-Aldrich Company. NaCl, MgCl_2_, KCl, HCl, and Tris were purchased from Beijing Chemical reagent company (Beijing, China). Whey protein powder (purity, 90%) was purchased from Fonterra Co-operative Group (Auckland, New Zealand). The enhanced BCA protein concentration assay kit was purchased from Beyotime Biotechnology (Shanghai, China). The 21-mer aptamer specific for kanamycin with *K*_*d*_ of 78.8 nM labeled with fluorophore (5′-FAM- TGG GGG TTG AGG CTA AGC CGA-3′, denoted as FDAN,) as described in the reference (Song et al., 2011), and three complementary strands labeled with quencher (denoted as QDNA: QDNA1, 3′-Dabcyl-ACC CCC A-5′; QDNA2, 3′-Dabcyl-ACC CCC AA-5′; QDNA3, 3′-Dabcyl-ACC CCC AAC-5′) were all synthesized by Shanghai Sangon Biological Engineering Technology & Services Co., Ltd. (Shanghai, China) and purified by HPLC. The solutions of FDNA and QDNA were all prepared with purified water, and stored at −20°C before use. Kanamycin stock solution was prepared with purified water, and stored at 4°C before use. The Tris-HCl buffer solution (pH 8.0) contained 150 mM NaCl, 5 mM KCl, and 1 mM MgCl_2_. The milk samples were purchased from the local supermarket in Changchun, China. All chemical reagents were analytical grade and the water used throughout all experiments was purified by a Milli-Q system (Millipore, Bedford, MA, USA).

### Apparatus

The CR20B2 refrigerated centrifuge (Hitachi, Japan) was used to perform centrifugation. The PHS-3C pH meter (Chenhua, China) was used to adjust buffer pH. The ultrasonication was performed by 125KQ-300DE ultrasonicator (Kunshan Ultrasonic Instrument Co., Shanghai, China). The fluorescence spectra was recorded by RF-5301 fluorescence spectrophotometer (Shimadzu, Tokyo, Japan) with excitation at 490 nm and slits for both the excitation and the emission were set at 5 nm. The WH-3 vortex mixer (MX-S, Shanghai, China) and the thermostat water bath (DK-8D) were also used during the experiment. The ACQUITY TQD UPLC-MS/MS (waters, America) was used for kanamycin detection in the contrast experiments according to the Chinese national standard methods.

### Establishment of Aptamer-Based Structure Switch

The aptamer-based structure switch in this study was composed of two parts, FDNA and QDNA. The FDNA and QDNA were diluted to 0.5 and 1 μM, respectively, heated in a thermostat water bath (95°C, 10 min), then cooled to room temperature for further use. In the 1.5 mL centrifugal tube, 50 μL FDNA (0.5 μM, final concentration 50 nM), 50 μL QDNA (1 μM, final concentration 100 nM), 350 μL Tris-HCl buffer (pH 8.0) and 50 μL water were added to obtain the ensemble solution. After the solution incubated at room temperature for 2 min, the fluorescence signal was measured. In this study, a series of experiments with different temperature variations were performed to verify the formation of structure-switching aptamers. In each group of experiment, the total reaction time between FDNA and QDNA at three stages of different temperature was 60 min. In the first stage, the solutions were incubated at 18°C for 10 min. In the second stage, the temperature was raised to a designated temperature (37, 45, 50, 55°C, respectively) within 5 min, and solutions were incubated for 30 min at raised temperatures. In the final stage, the solutions were cooled to 20°C within 5 min and incubated at 20°C for 10 min. During the whole temperature change process, the fluorescence signal was measured every 5 min to monitor the structure switching process of FDNA/QDNA duplex. Each experiment was carried out in triplicate. Unless otherwise specified, the final concentrations of FDNA and QDNA mentioned above were used in all the experiments.

### Structure-Switching Aptamers for Kanamycin Detection

Both of the FDNA (0.5 μM) and QDNA (1 μM) were heated at 95°C for 10 min in the thermostat water bath, then cooled down to room temperature before use. For the quantitative measurement of kanamycin, 50 μL different concentration of kanamycin was allowed to incubate with 50 μL 0.5 μM FDNA and 350 μL Tris-HCl buffer (pH 8.0) for 20 min. Next, 50 μL 1 μM QDNA was added and mixed for 2 min, with a final reaction volume of 500 μL. Fluorescence intensity of the reaction system was measured, and the standard curve was established with kanamycin concentration as abscissa and the increased fluorescence (*F–F*_0_) as ordinate, in which *F*_0_ and *F* were, respectively, the fluorescence intensity in the absence and presence of kanamycin. Each experiment was carried out in triplicate.

### Fluorescence Detection of Kanamycin in Milk Samples

Milk samples were processed in accordance with the published work (Ping et al., 2012). Firstly, 2 mL milk, 3 mL water, 1 mL 10% trichloroacetic acid, and 1 mL chloroform were added into a centrifuge tube in order and vortex-mixed for 1 min. Chloroform and trichloroacetic acid were used to deposit protein and dissolve organic substances in milk. Then, the mixture was processed in the ultrasonic instrument at 20°C for 15 min and centrifuged at 13,000 rpm for 10 min to separate the deposit. The supernatant was centrifuged again at 10,000 rpm for 10 min. Different concentrations of kanamycin were spiked into the milk, then pretreated and analyzed in accordance with the above procedure. Finally, kanamycin in the obtained supernatant was detected according to the assay of structure-switching aptamer. Each experiment was performed in three replicates.

### LC-MS/MS Detection of Kanamycin in Milk Samples

The spiked milk samples were also analyzed by LC-MS/MS for comparison to verify the reliability of the proposed assay based on structure-switching aptamers. The pretreatment of milk sample was carried out according to the Chinese National Standard GB/T 22969-2008 (Determination of streptomycin, dihydrostreptomycin and kanamycin residues in milk and milk powder LC-MS/MS method). Eight grams of milk and 30 mL 5% phosphoric acid solution were mixed for 10 min, then added 3 mL trichloroacetic acid. The mixture was thoroughly vortex-mixed and centrifuged at 4,000 r/min for 4 min. The supernatant was successively purified with the solid phase extraction columns of benzenesulfonate and carboxylic acid. The purified sample solution was filtered through the filter membrane (0.2 μm) and then the LC-MS/MS was tested. Liquid chromatography used Atlantis C_18_ chromatographic column (3.5 μm, inner diameter: 150 × 2.1 mm), the column temperature was 40°C, and the sample intake was 30 μL. The mobile phase A was 0.1% aqueous formic acid, mobile phase B was 0.1% acetonitrile formate and mobile phase C was methanol. Mass spectrometry conditions were electrospray ion source (ESI), positive ion scanning and multi-reaction monitoring (MRM). The electrospray voltage, the auxiliary gas flow rate, auxiliary gas temperature and the focusing voltage were 5 V, 7 L/min, 550°C, and 150 V, respectively. Two ion pairs at 485/163 and 485/324 of *m*/*z* were used for qualitative analysis and external standard method for quantitative analysis.

## Results and Discussion

### Mechanism Verification of Structure-Switching Aptamers

**Figure 1:**
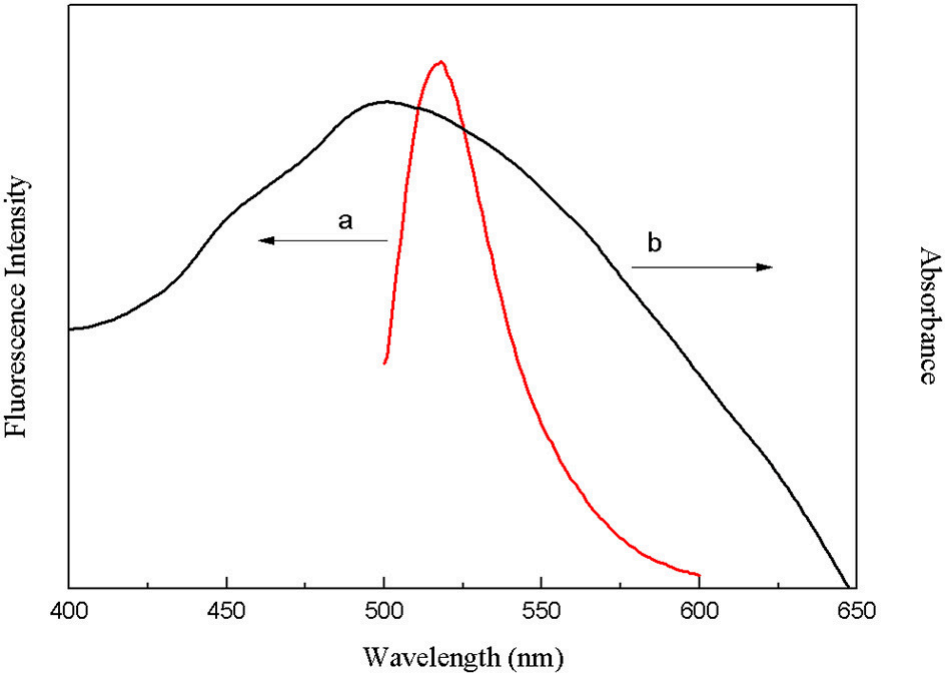
Overlap between the fluorescence emission spectrum of FAM fluorophore (a) and the absorption spectrum of Dabcyl quencher (b).

**Figure 2:**
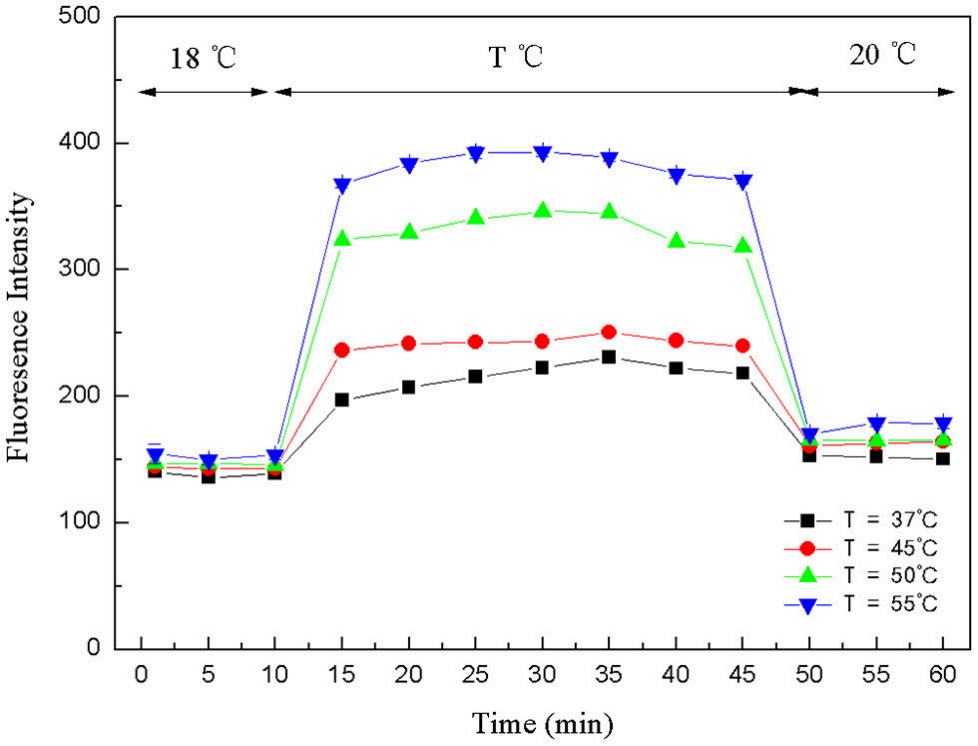
Fluorescence intensity changes of structure-switching aptamers at different temperatures. FDNA, 0.05 μM; QDNA, 0.1 μM.

The crucial premise for the establishment of a successful method based on structure-switching aptamers for kanamycin detection was to form an effective structure switch. This structure switch was composed of a FAM fluorophore-labeled kanamycin-binding aptamer (FDNA) and a short oligonucleotide (QDNA) modified with a Dabcyl quencher. When FDNA and QDNA formed the double helix structure with the spiral diameter of about 2 nm, the distance between the FAM fluorophore and the Dabcyl quencher was close enough to initiate FRET. And as shown in Figure 1, the emission spectrum of FAM fluorophore is obviously overlapped with the absorption spectrum of the Dabcyl quencher, suggesting that FRET can take place with FAM as donor and Dabcyl as acceptor. Thus, if FDNA and QDNA formed the double helix structure, the fluorescence emission of FAM fluorophore was drastically quenched. As is well-known that temperature is an important condition for denaturation and renaturation of DNA double helix structure, high temperature will break the hydrogen bonds to open the double helix structure, and cause the denaturation of DNA. After annealing, the denatured double-stranded DNA will return to the natural double helix structure. Therefore, a series of temperature-changing experiments were performed to verify the structure switching and the occurrence of FRET, the results were shown in Figure 2. Incubated at 18°C from 0 to 10 min, the structure switch had low and stable fluorescence intensity. Because of the formation of a stable double-stranded structure of FDNA and QDNA, the fluorophore and the quencher were close to each other, causing the fluorescence signal decreased. When the time was over 15 min, the temperature reached to the designed temperature, 37, 45, 50, or 55°C, and further incubation was continued at the raised temperature for 30 min. The fluorescence intensity was enhanced with the increased temperature from 37 to 55°C, because more and more QDNA was dissociated from the double-stranded structure. When the time reached 50 min, the reaction temperature was lowered to 20°C, free QDNA recombined with free FDNA to form a stable structure switch, the fluorescence intensity dropped. The above experimental results could verify the formation of the effective structure switch.

### Structure-Switching Aptamers Assay for Kanamycin Detection

**Figure 3:**
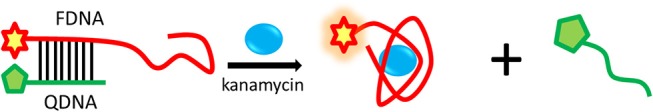
Schematic illustration of the structure-switching aptamers assay for kanamycin detection based on the FRET.

**Figure 4:**
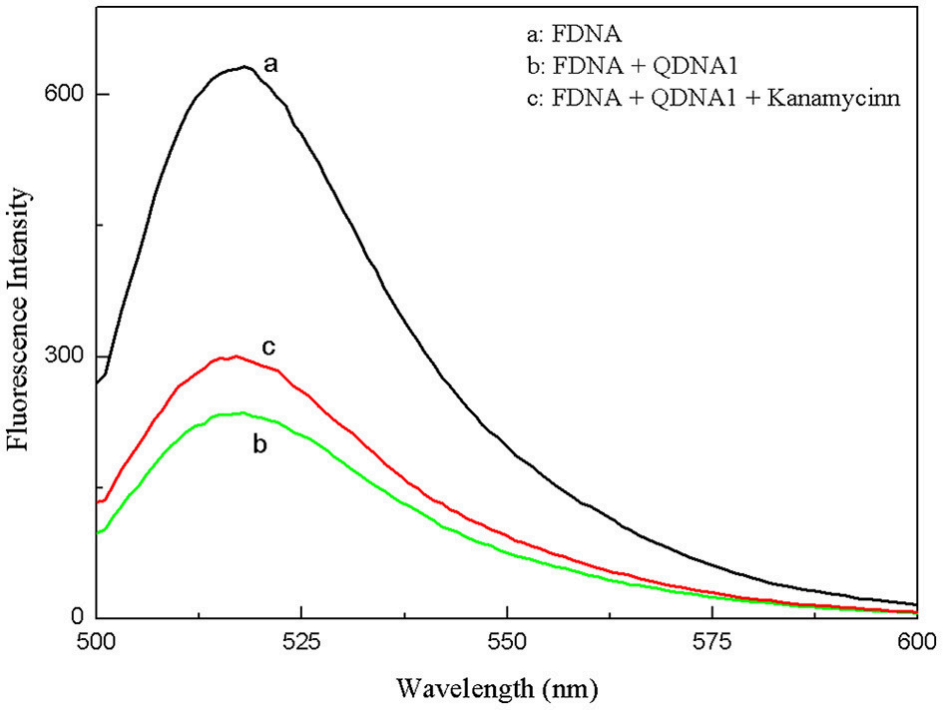
Feasibility experiments of the structure-switching aptamers assay for kanamycin. (a) FDNA, (b) FDNA + QDNA1, (c) FDNA + QDNA1 + kanamycin. FDNA, 0.05 μM; QDNA1, 0.1 μM; kanamycin, 500 nM.

The proposed strategy of structure-switching aptamers for kanamycin was based on the target-induced structure switching between FDNA/QDNA duplex and FDNA/target complex, and the principle was outlined in Figure 3. In this experimental system, kanamycin-binding aptamer was labeled with fluorophore (FDNA), and the complementary strand was labeled with quencher (QDNA). Without the target, the FDNA naturally bound to the QDNA and formed the double helix structure, bringing the fluorophore and the quencher into close proximity for effective fluorescence quenching due to the FRET mechanism. With the presence of target, the aptamer preferred to form the FDNA-target complex rather than FDNA/QDNA duplex due to the high affinity between aptamer and target. The FRET efficiency between FDNA and QDNA was decreased, resulting in the enhancement of fluorescence intensity. According to the Figure 4, when the excitation wavelength was 490 nm, the FDNA had a strong fluorescence (Figure 4, curve a) at the emission wavelength of 517 nm. Without kanamycin, FDNA hybridized to QDNA, and the structure switch was formed with the fluorescence signal quenched because of the FRET (Figure 4, curve b). When kanamycin was introduced, the fluorophore-labeled aptamers specifically bound to the target, and the QDNA was dissociated, thereby the fluorescence intensity was enhanced (Figure 4, curve c). The results from experimental spectrogram were consistent with the schematic diagram (Figure 3), so it can be verified that this experiment had certain feasibility.

### Optimization of Experimental Conditions

The optimization of reaction conditions was especially important for the detection system of kanamycin based on the structure switch. Therefore, conditions such as the base sequence of the complementary strand QDNA, the concentration ratio of FDNA and QDNA, the formation time of the structure switch, the reaction time of kanamycin and the aptamers, and the pH-value of the buffer solution, were optimized to establish the optimal detection method. Each experiment was replicated by three times.

#### Optimization of QDNA Base Number, and Concentration Ratio Between FDNA and QDNA

**Figure 5:**
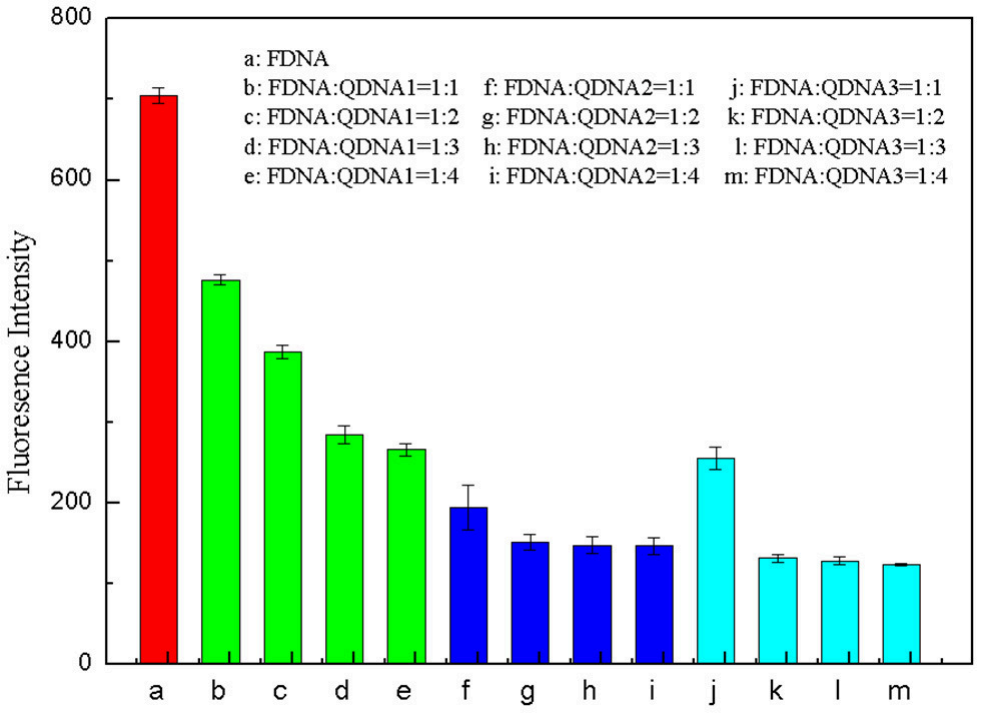
Optimization of concentration ratio between FDNA and QDNA. (a) FDNA, (b~e) FDNA: QDNA1 = 1:1~1:4, (f~i) FDNA: QDNA2 = 1:1~1:4, (g~m) FDNA: QDNA3 = 1:1~1:4. The concentration of FDNA was 0.05 μM, and QDNAs were 0.05, 0.1, 0.15, 0.2 μM, respectively.

The base sequence of the complementary strand QDNA was a key factor for the whole structure-switching system. QDNA should have an appropriate length, which can not only provide a low background of fluorescence intensity for the system when there is no target, but also can be released quickly after adding the targets. Therefore, three complementary strands (QDNA1, QDNA2, and QDNA3) with sequence length of 7, 8 and 9 bases were selected in this experiment to optimize the concentration ratio of the FDNA and the QDNA. The concentration of FDNA was diluted to 0.5 μM, and the QDNA was diluted to 0.5, 1, 1.5, and 2 μM. They were heated at 95°C for 10 min and naturally cooled to room temperature. The FDNA (50 μL) and the QDNA (50 μL) were mixed in Tris-HCl buffer solution (350 μL), and then the water (50 μL) was added to the reaction system. After being mixed for 15 min, the fluorescence signal was measured. The concentration ratio of FDNA and QDNA were set at 1:1, 1:2, 1:3, and 1:4. The results were shown in Figure 5, taking the concentration ratio as the abscissa and the fluorescence signal at 517 nm as the ordinate. As the sequence length of QDNA increased, the fluorescence signal decreased. As the concentration of the QDNA increased, the fluorescence signal also decreased, but it tended to be stable after decreasing to a certain extent. The longer sequence of the QDNA resulted in the stronger binding force with the FDNA, so the fluorescence background of the structure switch dropped more. The excessive QDNA could be combined with the FDNA to reach the saturated state, so the fluorescence signal did not change even if the QDNA concentration further increased. By comparing the quenching effect of QDNA in 12 groups of structure switch, QDNA2 was selected as the optimal base sequence, and the optimal concentration ratio between the FDNA and QDNA2 was 1:2 (Figure 5, column g).

#### Optimization of Structure Switch Incubation Time

**Figure 6:**
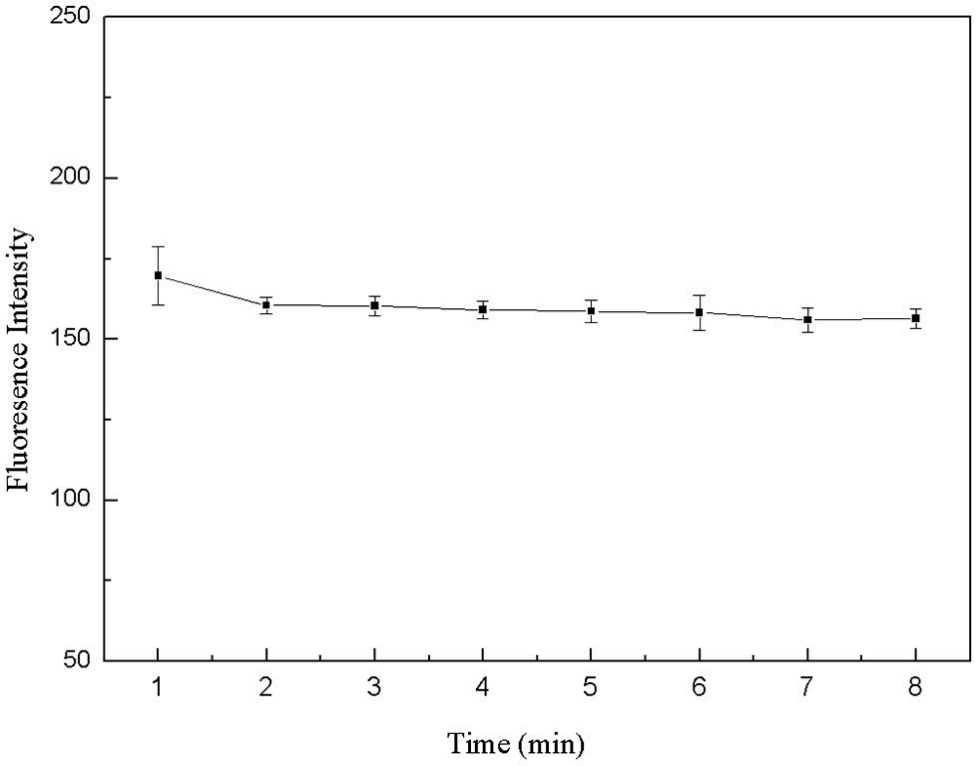
Optimization of formation time for the double-helix of structure switch. FDNA, 0.05 μM; QDNA2, 0.1 μM.

The 50 μL FDNA, 50 μL QDNA2, 350 μL Tris-HCl buffer solution and 50 μL purified water were added to the 1.5 mL centrifuge tube and mixed fully. The fluorescence intensity was measured every 1 min, as shown in Figure 6. At first, the fluorescence intensity decreased slightly, and after 2 min, it was basically stable. Therefore, the structure switch can be formed in a short time, and 2 min is selected as the optimal incubation time.

#### Optimization of Reaction Time for Kanamycin and Aptamer

**Figure 7:**
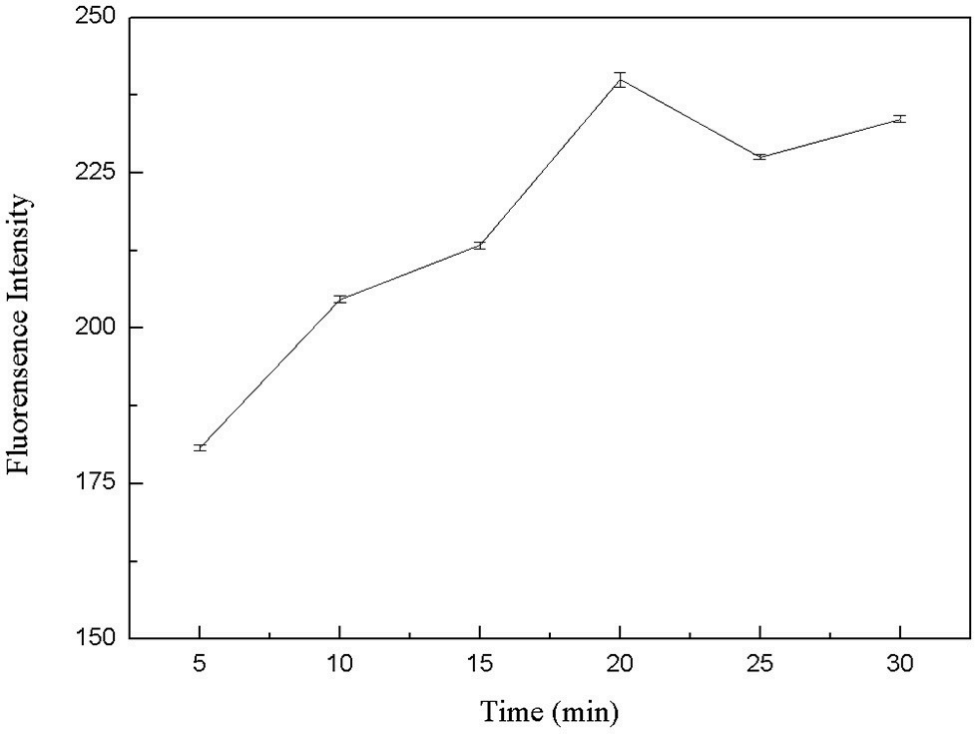
Optimization of reaction time between kanamycin and FDNA. FDNA, 0.05 μM; QDNA2, 0.1 μM; kanamycin, 500 nM.

The specific binding of kanamycin and aptamer was also a critical factor for the whole experimental system. Therefore, the binding time between kanamycin and aptamers was optimized. In six centrifuge tubes, 50 μL FDNA, 50 μL kanamycin, and 350 μL Tris-HCl buffer solution were added. They were mixed and incubated for 5, 10, 15, 20, 25, and 30 min, respectively. Then, QDNA2 was added and incubated for 2 min. The results from fluorescence measurements were shown in Figure 7. As the reaction went on, the aptamer and kanamycin gradually combined and QDNA2 were dissociated, resulting in the fluorescence signal intensity increased. When the reaction time was 20 min, the recovery of the fluorescence intensity was the highest, so the optimal reaction time was selected as 20 min.

#### Optimization of pH-Value for Tris-HCl Buffer Solution

**Figure 8:**
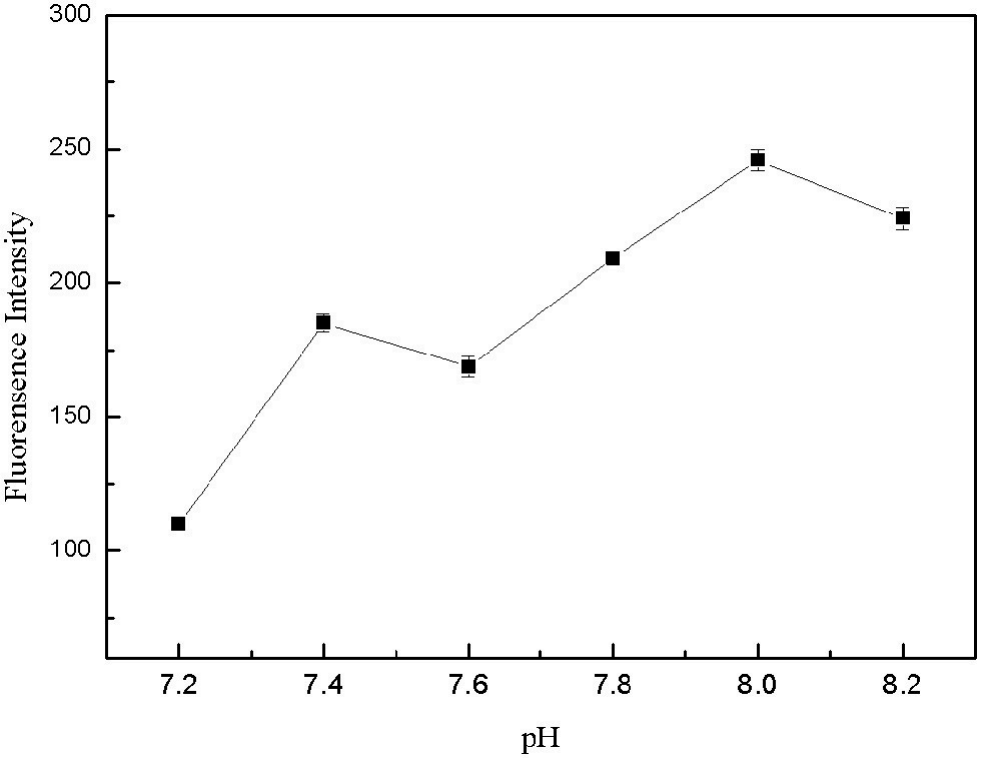
Optimization of pH-value for Tris-HCl buffer solution. FDNA, 0.05 μM; QDNA2, 0.1 μM; kanamycin, 500 nM.

The pH-value of the buffer solution affected the formation of the structure switch and the binding between the target and aptamers. Tris-HCl buffer solutions with different pH-value (7.2, 7.4, 7.6, 7.8, 8.0, 8.2) were prepared, which contained 150 mM NaCl, 5 mM KCl and 1 mM MgCl_2_. 50 μL FDNA, 50 μL kanamycin and 350 μL Tris-HCl buffer solution with different pH-value were mixed for 20 min, then QDNA2 was added and incubated for 2 min, finally the fluorescence intensity of the detection system was measured. As shown in Figure 8, within the pH range of 7.2–8.2, the highest fluorescence intensity was obtained at the pH of 8.0. Therefore, the Tris-HCl buffer solution with pH of 8.0 was used in this system.

### Analytical Performance

The fluorescence intensity of the structure-switching aptamer system was measured in the presence of different concentrations of kanamycin under the optimal reaction conditions. The final concentrations of the kanamycin were 100, 200, 300, 400, 500, 600, 700 nM. The standard curve of the system was established by taking the concentration of kanamycin as abscissa and the recovery value of fluorescence signal (*F*–*F*_0_) as the ordinate, in which *F*_0_ was the fluorescence intensity without the kanamycin and *F* was the fluorescence intensity with the presence of kanamycin. With the increment of kanamycin concentration, the fluorescence intensity of the system gradually increased, and there is a good linear relationship between kanamycin concentration and the fluorescence signal recovery. The linear range of the method was 100–700 nM with the detection limit of 12.0 nM (*3*σ), which is well below the MRL of kanamycin in milk, demonstrating that this method could be used for the detection of traces of kanamycin in real samples (Data not shown).

**Figure 9:**
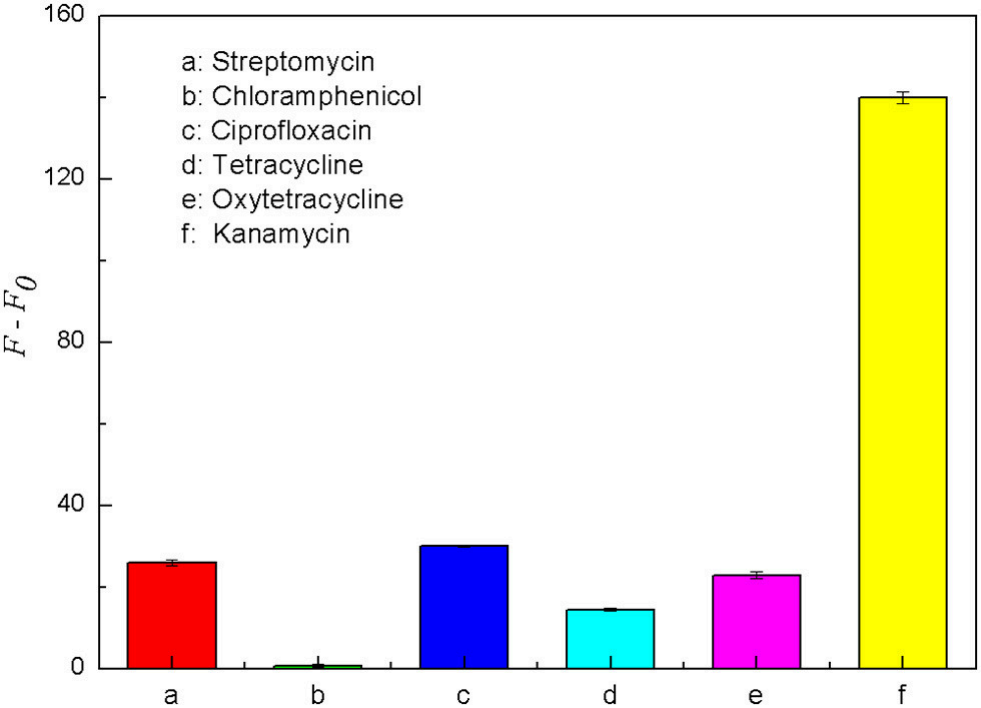
Comparison of the fluorescence recovery (*F–F*_0_) of this assay used for different antibiotics, including streptomycin (a), chloramphenicol (b), ciprofloxacin (c), tetracycline (d), oxytetracycline (e) and kanamycin (f). The concentration of FDNA was 0.05 μM, QDNA2 was 0.1 μM, and the antibiotics were 500 nM.

Some antibiotics are similar to kanamycin in structure and property, and they may interfere with the specific detection of kanamycin in milk samples. Therefore, five common antibiotics including tetracycline, oxytetracycline, streptomycin, chloramphenicol and ciprofloxacin were selected for the specificity analysis. Each of these antibiotics was prepared into the solution with the final concentration of 500 nM. As shown in Figure 9, when the target was kanamycin, the fluorescence recovery of the detection system was significantly higher than the other five antibiotics. The results indicated that the fluorescence intensity could be significantly recovered only in the presence of kanamycin. It means that only kanamycin could bind better with its aptamer and effectively disrupt the double helix structure of the structure switch. Therefore, the aptamer-based structure switching established in this experiment had a good specificity for kanamycin, which can be used to detect kanamycin in complex sample matrixes.

### Analytical Application to Milk Samples

**Figure 10:**
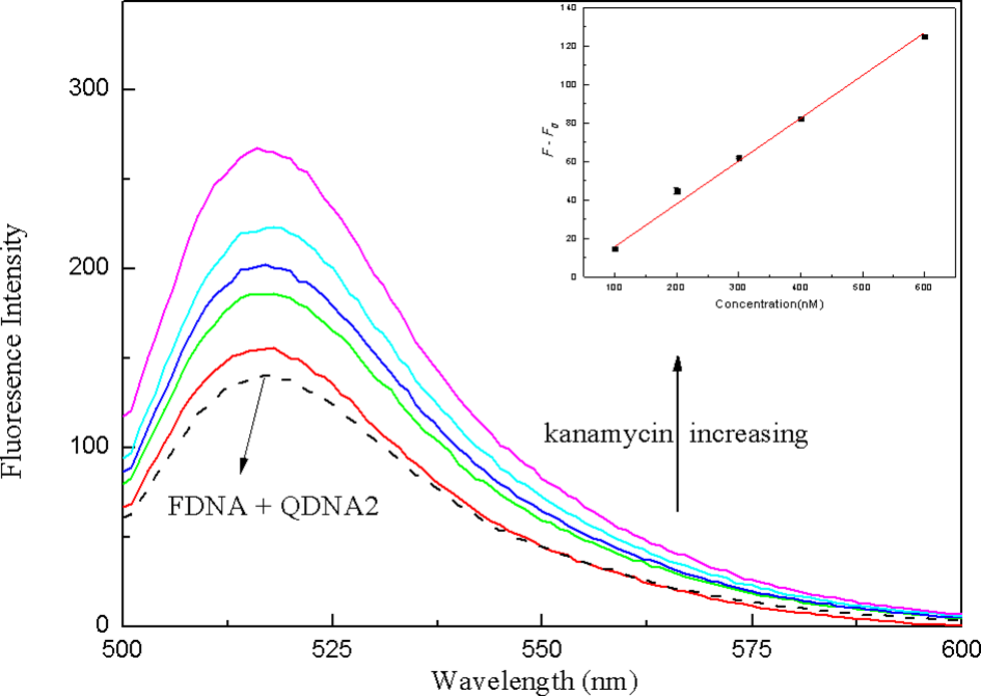
The fluorescence spectra of the structure-switching aptamers for different concentrations of kanamycin spiked in milk samples. Inset: the linear calibration of fluorescence recovery (*F–F*_0_) vs. kanamycin concentration in milk samples. The concentration of FDNA was 0.05 μM, QDNA2 was 0.1 μM, and kanamycin was 100, 200, 300, 400, 600 nM, respectively.

In order to verify the practical application of the structure-switching aptamers assay, the standard addition method was used to detect kanamycin in milk samples. Firstly, as shown in Table 1, the potential interfering substances in milk samples (http://en.wikipedia.org/wiki/Milk#Nutritional_vaule) were evaluated to ensure that the established method was applicable to the detection of kanamycin in real milk samples. The results showed that for the detection of 500 nM kanamycin, the changes in fluorescence intensity caused by the presence of interfering substances are all < ±5%. It indicated that the coexisting constituents could hardly produce obvious interferences for kanamycin detection. The protein in the pretreated sample was measured using the enhanced BCA protein concentration assay kit, and it was found that the protein residue in the solution was about 700 μg/mL. Normal bovine milk contains 30–35 g/L protein of which about 80% is arranged in casein micelles and 20% is whey protein (https://en.m.wikipedia.org/wiki/Milk). Since the pH of the milk sample solution is lowered after treating with trichloroacetic acid, the casein micelles are destroyed, and casein and whey protein are precipitated (Liu and Guo, 2008; Ouanezar et al., 2012; Silva et al., 2013). Casein is insoluble in water and organic solvents (https://en.m.wikipedia.org/wiki/Casein), so the pretreated sample solution can be considered to be almost free of casein. The residual protein in the sample is water-soluble whey protein. Therefore, the whey protein interference test was performed on the detection system, and the results showed that the 850 μg/mL of whey protein changed the fluorescence intensity to −1.65%, which was considered to be non-interference. Then, the milk was pretreated according to the procedures described in the section Materials and Methods, and the spiked samples with final kanamycin concentrations of 100, 200, 300, 400, 600 nM were prepared and analyzed. The results were shown in Figure 10, with the increasing of kanamycin concentration, the fluorescence intensity of the system was gradually recovered in the linear range from 100 to 600 nM, with the detection limit of 13.52 nM (*3*σ). Compared with the published aptasensors for kanamycin listed in Table 2, the detection limit is not the lowest, but it is significantly lower than the MRL in milk. Therefore, the proposed structure-switching aptamers assay is applicable to the detection of kanamycin in milk, and it exhibits the merits of simple operation and rapid analysis. The affinity of aptamer and target may be further improved by splitting the aptamer of target (Zou et al., 2012; Zhao et al., 2015).

**Table 1:** The influences of various potential interfering substances on the fluorescence intensity of the detecting system.

**Interfering substances**	**Concentration in milk (g·L^**−1**^)**	**Added concentration** **(g·L^**−1**^)**	**Tolerance ratio**	**Change of fluorescence intensity (%)**
K^+^	1.36	13.60	10	4.48
Na^+^	0.443	26.58	60	4.42
Mg^2+^	0.103	10.30	100	−5.01
Cl^−^	1.07	10.70	10	4.83
NO${}_{3}^{-}$	0.158	7.90	50	−4.67
Ca^2+^	1.16	58.00	50	−4.69
Vitamin B_1_	0.000474	0.00948	20	−4.42
Vitamin B_2_	0.00183	0.0366	20	−4.07
Vitamin B_12_	0.0000045	0.0045	1,000	4.13
Vitamin C	0.0237	0.474	20	−4.12
L-lysine	2.72	27.20	10	4.24
L-tryptophan	0.412	10.30	25	4.01
L-threonine	1.38	138.00	100	4.36
Glycine	0.639	63.90	90	−4.71
L-histidine	0.979	48.95	50	−4.21
Glucose	0.186	55.80	300	−4.39
Lactose	52.00	260.00	5	4.92

*FDNA, 0.05μM; QDNA2, 0.1μM; kanamycin, 500nM*.

**Table 2:** Comparison of various kanamycin detection methods using kanamycin-binding aptamer.

**Methods**	**Linear range (nM)**	**Detection limit (nM)**	**Matrix**	**References**
Electrochemical aptasensor	0.1–100	0.0745	Milk	Li et al., 2018
Strip biosensor	1–30	0.0778 100 (visual limit)	Milk	Liu et al., 2018
Evanescent wave aptasensor	200–200,000	26	Milk	Tang et al., 2018
Electrochemical impedance spectroscopy (EIS) based aptasensor	2.06–128.73	0.188	Milk	Sharma et al., 2017
Label-free fluorescent aptasensor	24.75–137.15	7.5	Serum	Dehghani et al., 2018
Structure-switching aptamers assay	100–600	13.52	Milk	This work

In order to verify the reliability of the established method in application of actual samples, the milk samples spiked with different concentrations of kanamycin were analyzed by this method and LC-MS/MS (the Chinese National Standard GB/T 22969-2008) for comparison. As shown in Table 3, the recoveries of this established method was 101.3–109.1% with the relative standard deviation of 0.4–1.1%, consistent with the results from LC-MS/MS. The results indicated that the established structure-switching aptamers assay is highly reproducible and accurate for rapid detection of kanamycin. Furthermore, including sample pretreatment procedure, the whole test process could be completed within 1 h, which can meet the rapid detection for kanamycin.

**Table 3:** Results for the detection of different amounts of kanamycin spiked in milk by this established method and LC-MS/MS (*n* = 3).

**Sample**	**Added** **(nM)**	**Found (nM)**		**Recovery (%)**	
		**This method**	**LC-MS/MS**	**This method**	**LC-MS/MS**
1	400	413	398	103.4 ± 0.8	99.5
2	500	506	463	101.3 ± 0.4	92.8
3	600	655	629	109.1 ± 1.1	104.8

## Conclusion

In this work, a structure-switching aptamers assay for the detection of kanamycin was established by using the highly specific recognition of kanamycin-binding aptamer and the sensitive FRET between FDNA and QDNA. Under optimal conditions, this structure-switching aptamers assay was successfully applied to detect kanamycin in milk samples, with the detection limit as low as 13.52 nM. The whole procedure including sample pretreatment was convenient to operate and could be accomplished within 1 h, which can satisfy the rapid detection for kanamycin in real samples. In summary, this structure-switching aptamers assay has the advantages of low cost, simple operation, rapid detection, good selectivity, and high sensitivity. Moreover, due to the design of the aptamer-based structure switch was simple and universal, this method could be applied for the detection of other targets by changing the aptamers without learning the secondary or tertiary structure of the aptamer. This method can also be extended to the detection of multiple targets. Since the method is based on the FRET principle, homogeneous detection of multiple targets can be achieved by labeling different fluorophores and quenchers on structure switch of different targets.

## Author Contributions

XM, SQ, HS, MZ, and CS conceived the idea and designed the experiments. XM, SQ, and CS designed and manufactured structure switch. XM, SQ, HS, and RS performed experiments. XM, SQ, HS, RS, MZ, and CS contributed to data analysis and interpretation. XM and CS wrote this paper. All authors discussed the results and commented on the manuscript.

### Conflict of Interest Statement

The authors declare that the research was conducted in the absence of any commercial or financial relationships that could be construed as a potential conflict of interest.
